# Diaphragmatic paralysis, respiratory function, and postoperative pain after interscalene brachial plexus block with a reduced dose of 10 ml levobupivacaine 0.25% versus a 20 ml dose in patients undergoing arthroscopic shoulder surgery: study protocol for the randomized controlled double-blind REDOLEV study

**DOI:** 10.1186/s13063-021-05216-6

**Published:** 2021-04-19

**Authors:** P. Oliver-Fornies, J. P. Ortega Lahuerta, R. Gomez Gomez, I. Gonzalo Pellicer, L. Oliden Gutierrez, J. Viñuales Cabeza, L. Gallego Ligorit, C. E. Orellana Melgar

**Affiliations:** 1 Morphological Madrid Research Center Investigator, Department of Anesthesiology, Critical Care and Pain Management, Lozano Blesa University Clinical Hospital, Aragon Institute for Health Research, Avda. San Juan Bosco, 15 50009 Zaragoza, Spain; 2 Division of Regional Anesthesia, Department of Anesthesiology, Critical Care and Pain Management, Miguel Servet University Hospital, Zaragoza, Spain; 3 Department of Anesthesiology, Critical Care and Pain Management, Miguel Servet University Hospital, Aragon Institute for Health Research, Zaragoza, Spain; 4 Department of Pneumology, Miguel Servet University Hospital, Zaragoza, Spain

**Keywords:** Diaphragmatic paralysis, Interscalene brachial plexus block, Ultrasound, Spirometry, Postoperative pain, Arthroscopic shoulder surgery, Randomized controlled trial

## Abstract

**Background:**

Arthroscopic shoulder surgery causes severe postoperative pain. An interscalene brachial plexus block provides adequate analgesia, but unintended spread of the local anesthetic administered may result in a phrenic nerve block, usually associated with a nonnegligible incidence of acute hemidiaphragmatic paralysis.

The main purpose of this trial will be to analyze the incidence of hemidiaphragmatic paralysis ensuing after interscalene brachial plexus block in patients undergoing arthroscopic shoulder surgery administered a standard volume (20 ml) vs. a low volume (10 ml) of levobupivacaine 0.25%.

**Methods:**

This will be a prospective double-blind randomized controlled single-center two-arm comparative trial. Forty-eight patients will be included. The primary goal will be to ultrasonographically determine the incidence of hemidiaphragmatic paralysis by calculating the diaphragmatic thickness ratio in each group. The secondary goals will be to compare the two arms in terms of (1) decrease in forced vital capacity and (2) in forced expiratory volume at 1 s by spirometry; (3) decrease in diaphragmatic excursion by ultrasound; (4) 24-h total intravenous morphine consumption; (5) time to first opioid request of a patient-controlled analgesia pump; and (6) postoperative complications.

**Discussion:**

This trial will demonstrate that a low-volume interscalene brachial plexus block decreases hemidiaphragmatic paralysis following arthroscopic shoulder surgery according to spirometry and ultrasound measurements and does not provide inferior postoperative analgesia to the standard volume, as measured by opioid requirements.

**Trial registration:**

EudraCT and Spanish Trial Register (REec) registration number: 2019-003855-12 (registered on 7 January 2020). ClinicalTrials.gov identification number: NCT04385966 (retrospectively registered on 8 May 2020). *Ethics Committee* approval: EC19/093 (18 December 2019).

**Supplementary Information:**

The online version contains supplementary material available at 10.1186/s13063-021-05216-6.

## Administrative information

Note: the numbers in curly brackets in this protocol refer to SPIRIT checklist item numbers. The order of the items has been modified to group similar items (see http://www.equator-network.org/reporting-guidelines/spirit-2013-statement-defining-standard-protocol-items-for-clinical-trials/).

**Table:** 

Title {1}	Diaphragmatic paralysis, respiratory function, and postoperative pain after an interscalene brachial plexus block with a reduced dose of 10 ml levobupivacaine 0.25% versus a 20 ml dose in patients undergoing arthroscopic shoulder surgery: study protocol for the randomized controlled double-blind REDOLEV study.
Trial registration {2a and 2b}.	ClinicalTrials.gov NCT04385966. EudraCT 2019-003855-12. Available at: https://www.clinicaltrialsregister.eu/ctr-search/search?query=2019-003855-12
Protocol version {3}	(17 January 2021) Version 2.0
Funding {4}	This is investigator-initiated research with no source of funding.
Author details {5a}	P. Oliver-Fornies^1^, J.P. Ortega Lahuerta^2^, R. Gomez Gomez^2^, I. Gonzalo Pellicer^2^, L. Oliden Gutierrez^2^, J. Viñuales Cabeza^2^, L. Gallego Ligorit^3^, C.E. Orellana Melgar^4^. ^1^(Corresponding author): Department of Anesthesiology, Critical care and Pain management, Lozano Blesa University Clinical Hospital, GIIS083 Investigator of Aragon Institute for Health Research. Address: Avda. San Juan Bosco, 15 50009 Zaragoza, Spain. Phone: +34 976 76 57 00. E-mail address: pablo.oliver.fornies@gmail.com. ORCID ID: 0000-0001-7483-4807. ^2^Division of Regional anesthesia, Department of Anesthesiology, Critical care and Pain management, Miguel Servet University hospital, Zaragoza, Spain. ^3^Department of Anesthesiology, Critical care and Pain management, Miguel Servet University hospital, GIIS083 Investigator of Aragon Institute for Health Research, Zaragoza, Spain. ^4^Department of Pneumology, Miguel Servet University hospital, Zaragoza, Spain.
Name and contact information for the trial sponsor {5b}	Aragon Institute for Health Research *(*IISAragon). Centro de Investigación Biomédica de Aragón (CIBA). Avda. San Juan Bosco, 13. 50009. Zaragoza, Spain. Phone: +34 976 71 6818. email: info@iisaragon.es.
Role of sponsor {5c}	Monitoring

## Introduction

### Background and rationale {6a}

Arthroscopic shoulder surgery causes severe postoperative pain, which can compromise early patient rehabilitation. Clinical practice guidelines recommend a multimodal analgesic regimen including regional analgesic techniques such as interscalene brachial plexus block (IBPB: ICD-10 code 3E0T3CZ) in this surgery [1, 2]. IBPB offers adequate postoperative analgesia, a low incidence of nausea and vomiting, and low opioid requirements. However, it could also result in severe complications such as phrenic block [1]. In 1991, Urmey et al. found that 100% of their IBPBs produced a phrenic blockade with acute ipsilateral hemidiaphragmatic paralysis (HDP), which decreased pulmonary function (ICD-10 code: J98.6) [2–5]. The advent of ultrasound (US) has allowed a reduction in the doses of local anesthetic (LA) required in IBPB. Recent publications have shown a reduction in the incidence of HDP to 10–26% [6–9]. Diaphragmatic dysfunction can be detected as a decrease in forced vital capacity (FVC) and forced expiratory volume at 1 s (FEV1) on spirometry or as lower diaphragmatic excursion (DE) on US, the latter having become the gold standard in thoracic assessment [10].

Classically, performance of an IBPB has been contraindicated in patients with decreased pulmonary function [3]. As a result, some publications have tried to find a safe low LA IBPB dose that could decrease postoperative complications in these patients, while others have tried to find alternative regional anesthetic techniques. The fact is, however, that HDP remains a major concern and more randomized controlled trials are needed to warrant performance of IBPB with lower LA doses.

This article will describe the design and analytical protocol of the REDOLEV-2019 study (Reduction DOse of LEVobupivacaine Study), a randomized controlled comparative study designed to determine the incidence of HDP in patients undergoing an IBPB with a standard volume versus a low volume dose of levobupivacaine 0.25% during arthroscopic shoulder surgery. We hypothesized that reducing the volume of levobupivacaine 0.25% from the standard volume (20 ml) to a lower volume (10 ml) could decrease the incidence of HDP as measured both by US and spirometry, and that such lower volume would not be inferior in terms of postoperative pain or complications.

### Objectives {7}

The primary goal of this study is to determine the incidence of HDP following IBPB a 10 ml versus 20 ml volume of levobupivacaine 0.25% in patients undergoing arthroscopic shoulder surgery as measured by calculating the diaphragmatic thickness ratio (DTR) by US.

Secondary study objectives include a comparison of the incidence of HDP as measured by DE on US, and FVC, FEV1, and peak expiratory flow (PEF) on spirometry, as well as postoperative pain and complications after the performance of an IBPB with a 10 ml versus a 20 ml volume of levobupivacaine 0.25% during arthroscopic shoulder surgery.

### Trial design {8}

**Fig. 1:**
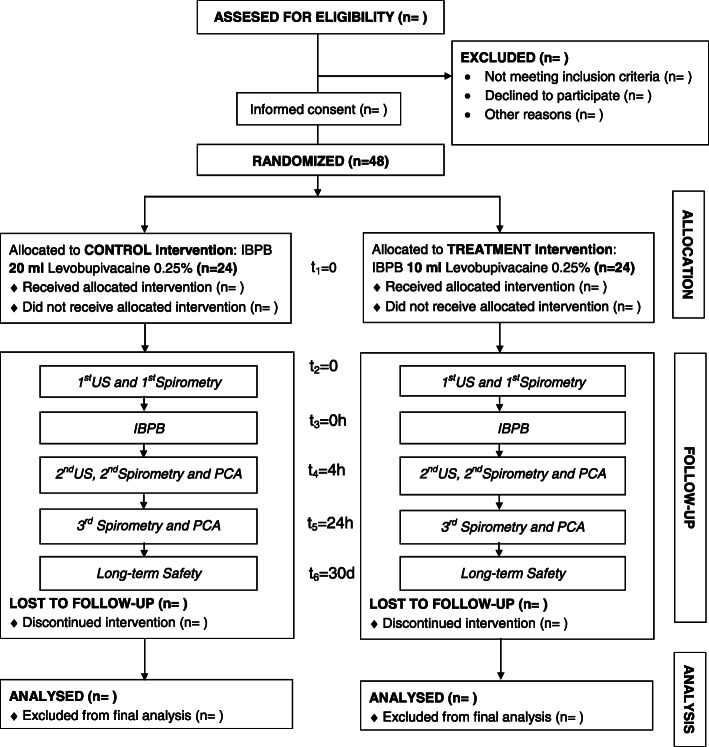
CONSORT diagram of the REDOLEV-2019 study participant flow [11]. Abbreviations: IBPB, interscalene brachial plexus block; PCA, patient-controlled analgesia; US, ultrasounds

The REDOLEV-2019 trial has been designed as a phase III randomized prospective double-blind single-center two-arm comparative controlled clinical trial (RCT). The present report has been drafted in accordance with the Consolidated Standards of Reporting Trials (CONSORT) and the SPIRIT statements [11, 12]. Attached to this report are the SPIRIT checklist protocol and the SPIRIT diagram of the study participant timeline. The study will include 48 patients undergoing elective arthroscopic shoulder surgery under general anesthesia with an IBPB. Eligible patients will be randomly allocated to one of 2 groups: a control group (G1), who will receive standard-volume IBPB (20 ml of levobupivacaine 0.25%), or a treatment group (G2), who will receive low volume IBPB (10 ml of levobupivacaine 0.25%). Figure 1 shows the trial flowchart.

## Methods: participants, interventions, and outcomes

This trial will be conducted in compliance with the European Union Clinical Trials Directive (2001/20/EC) and the principles of the Declaration of Helsinki (2013) [13, 14].

### Study setting {9}

Participants will be recruited exclusively from the Shoulder Surgery Division and Regional Anesthesia Division of the HUMS in Zaragoza, Spain.

### Eligibility criteria {10}

Eligible patients must comply with the following inclusion criteria at randomization: (1) aged from 18 to 80 years, (2) ASA I–III, and (3) scheduled for elective arthroscopic shoulder surgery and IBPB. The exclusion criteria will be as follows: (1) age < 18 and > 80 years; (2) pregnancy; (3) inability to undergo IBPB or spirometry; (4) allergy to the anesthetic drugs used in the study; (5) history of pulmonary disease (moderate or severe chronic obstructive pulmonary disease or severe asthma), diaphragmatic paralysis or neuromuscular disease or brachial neuropathy; (6) coagulation disorder; and (7) chronic opioid consumption: > 3 months or oral morphine equivalent to > 5 mg per day for a month.

### Who will take informed consent? {26a}

Written informed consent (IC) with impartial witnesses will be obtained from all participants by the principal investigator (PI) after hospital admission.

### Additional consent provisions for collection and use of participant data and biological specimens {26b}

By giving their IC, participants will agree to the storage of data and publication of the results in the main and ancillary studies. This trial does not involve collecting biological specimens for storage.

## Interventions

### Explanation for the choice of comparators {6b}

A review of the literature showed that LA regimens involving volumes greater than 10 ml and concentrations higher than 0.25% are associated with a high incidence of HDP [15–17]. Therefore, this study will use a low volume (10 ml of levobupivacaine 0.25%: 25 mg), which has been shown to offer adequate postoperative analgesia. As a comparator, it will use 20 ml of levobupivacaine 0.25% (50 mg), which is the one usually administered in our hospital. The safety of levobupivacaine is well known [18].

### Intervention description {11a}

Eligible participants will be randomized in equal proportions between two groups mentioned above, receiving their treatment only once as a single-shot block before surgery.

Spirometry and US will be performed at baseline and 4 and 24 h postoperatively. After admission to the post-anesthesia care unit (PACU), patients will be provided with an intravenously (IV) morphine patient-controlled analgesia (PCA) pump.

Interventions will be fully integrated within the hospital’s routine clinical practice.

The IBPB will be guided by US with a linear transducer (GE Medical, Milwaukee, WI, USA). After skin sterilization, an in-plane approach with a 22-gauge 50 mm Stimuplex® Ultra 360° needle (B. Braun, Melsungen, Germany), will be used to inject the study LA into the interscalene space at the C5-C6 level. This US-guided approach has been described by Nadeau et al. [19]. Before surgery, the efficacy of the block must be confirmed by pinprick testing. Evidence of block failure after 15 min will exclude the case from analysis.

Diaphragmatic US will be performed before (baseline) and 4 h (at least 1 h after extubation) after IBPB in a sitting and supine position. The ipsilateral and contralateral hemidiaphragms will be assessed using a linear US transducer (GE Medical, Milwaukee, WI, USA). The US apposition zone will be assessed in the anterior axillary line, and diaphragmatic thickness will be recorded to obtain the maximal inspiration (inspiratory diaphragmatic thickness) and expiratory (expiratory diaphragmatic thickness) values. The ratio between the two values constitutes the DTR. DE will be assessed on maximal inspiration and expiration, evaluating variations in the number of intercostal spaces and diaphragmatic motion (normal/caudal, null or cephalic/paradoxical).

Spirometry will be performed using a bedside spirometer (Air-Smart Spirometer; NuvoAir AB© 2020, Riddargatan 17D, SE-11457 Stockholm, Sweden) before (baseline) and 4 (at least 1 h after extubation) and 24 h after IBPB in the sitting and supine positions. It will be performed in accordance with the standards of lung function testing of the American Thoracic Society (ATS) and the Spanish Society of Pulmonology and Thoracic Surgery (SEPAR) [20–22]. FVC, FEV1, FEV1/FVC ratio and PEF will be measured three times at every assessment to comply with acceptable and reproducibility criteria. The best FVC and FEV1 effort will be recorded.

Combined Regional-General Anesthesia: Before general anesthesia induction, IBPB will be performed in the operating room. Antibiotic prophylaxis will be given IV. All patients will receive an IV general anesthesia with fentanyl 2 μg kg^−1^, propofol 2 mg kg^−1^, and rocuronium 0.6 mg kg^−1^ before being orotracheally intubated. Mechanical ventilation will be performed with the respiratory rate and tidal volume suitably adjusted to maintain normocapnia. The bispectral index (BIS) and train of four (ToF) will be measured. Anesthesia will be maintained with sevoflurane (0.7–1 MAC) to obtain a 40–60 BIS index. Intraoperative analgesia with IV remifentanil (0.05–0.2 μg min kg^−1^) will be administered. Every patient will receive 1 g IV paracetamol and 50 mg IV dexketoprofen, 30 min before extubation. Eight milligrams IV dexamethasone and 4 mg IV ondansetron will be given as anti-emetic prophylaxis. Fluid management with balanced crystalloids will be performed to maintain normovolemia. Before extubation, ToF 4/4 < 90% Sugammadex 2 mg.kg-1 will be used. After leaving the operating room, patients will be taken to the PACU.

Arthroscopic shoulder surgery will be routinely performed in the decubitus lateral position by the same two surgeons. No concurrent open repairs will be included.

A postoperative PCA pump (CADD®-Solis Infusion System, Smiths Medical, Minneapolis; USA) with IV morphine will be administered from admission to the PACU until 24-h postoperative. The PCA pump will be set for 1 mg bolus (2 ml) doses with a lockout time of 10 min without continuous infusion. Pain will be recorded on a numeric rating scale (NRS) (0–10) at the PACU and at 24 h.

PACU Interventions: The second US and spirometry analyses will be performed at the PACU. Participants will be discharged from the PACU after achieving an Aldrete score > 8/10.

Hospital Interventions: During hospitalization, all patients will receive 1 g IV paracetamol and 50 mg IV dexketoprofen alternatively every 4 h since they will discharge from PACU. IV metamizol (2 g) will be given to participants with an allergy to non-steroidal anti-inflammatory drugs. IV ondansetron (4 mg) will be given every 12 h as nausea and vomiting prophylaxis. After 24 h’ follow-up, the NRS score will be recorded. Follow-up will finish 30 days postoperatively after checking the incidence, frequency, and severity of serious adverse events (SAEs) and any hospital readmission history.

### Criteria for discontinuing or modifying allocated interventions {11b}

The main study intervention will be a single-shot IBPB. Once performed, the intervention cannot be discontinued or modified.

### Strategies to improve adherence to interventions {11c}

Before the study intervention, every participant will be trained to perform spirometry and manage a PCA. All study drugs will be provided and relabelled by the HUMS’ Pharmacy Department. After recruitment, the pharmacy staff will dispose of the packaging of drugs used during the trial according to standard disposal practice in accordance with the Ethics committee of clinical research of Aragon (CEICA) guidelines.

### Relevant concomitant care permitted or prohibited during the trial {11d}

Only analgesia allowed in the protocol will be administered to the participants.

### Provisions for post-trial care {30}

The study interventions are current routine anesthesia practice so no provision needs to be made in this regard.

### Outcomes {12}

The primary endpoint of the REDOLEV-2019 trial will be the incidence of HDP according to the US- determined DTR in the two trial groups. IBPB-induced HDP will be diagnosed if DTR < 1.2 [23, 24]. US will be performed before surgery and at 4 h after IBPB.

Secondary study endpoints will be (1) incidence of HDP diagnosed by spirometry, determined by measuring both FVC and FEV1 values at baseline and at 4 and 24 h post-op (HDP will be deemed to be present if a decrease ≥20% in FVC and (2) FEV1 values is observed); (3) incidence of HDP as manifested by US-determined DE in terms of a decrease in intercostal spaces (reduction ≥ 25%) and potential diaphragmatic motion (positive to paradoxical or null motion); (4) postoperative 24-h cumulative IV morphine consumption (mg); (5) time (min) to the first opioid request of the PCA pump; and (6) incidence, frequency and severity of (serious) adverse events according to CTCAE v5.0 [25]. Every secondary endpoint will be compared between the study groups. This is a per intention-to-treat study.

The time points for measurement of the primary and secondary endpoints are detailed in Table 1.

**Table 1:** Participant timeline of REDOLEV-2019 Clinical Trial

	Study period				
	Enrolment	IBPB	Post-allocation		Close-out
Timepoint	Before surgery	0	4 h	24 h	30 days
**Enrolment:**					
**Eligibility screen**	**X**				
**Informed consent**	**X**				
**Medical background**	**X**				
**Random Allocation**	**X**				
**Interventions:**					
**G1: IBPB 20 ml**		**X**			
**G2: IBPB 10 ml**		**X**			
**Assessments:**					
**US: DTR, ED (n°esp) and ED (type)**	**X**		**X**		
**Spirometry (FVC and FEV1)**	**X**		**X**	**X**	
**Pain: 24 h total consumption and time to first request.**			**X**	**X**	
**Complications**			**X**	**X**	**X**

This template is copyrighted by the SPIRIT Group. Abbreviations: *DE* diaphragmatic excursion, *DTR* diaphragmatic thickness ratio, *FVC* forced vital capacity, *FEV1* forced expiratory volume at 1 s, *IBPB* interscalene brachial plexus block

### Participant timeline {13}

The schedule diagram of the participant’s timeline is shown in Table 1.

### Sample size {14}

Drawing on previous studies, this trial will assume an IBPB-induced HDP rate of 90% for the control group and of 33% for the treatment group [6, 15, 26]. These findings suggest that decreasing the IBPB LA dose (10 ml, 0.25%, or 25 mg) should result in less IBPB-induced HDP.

Therefore, powering this trial to identify a mean difference of 90–33% with a two-sided significance level of 1% and a power of 90% with the same allocation to the two arms will require 21 patients in each arm. Assuming a 10% dropout rate, 24 patients will be enrolled per arm, i.e., 48 participants will be the total sample size, as calculated using Epidat® software [27, 28].

### Recruitment {15}

HUMS provides health care to a population of more than 350,000 inhabitants, mostly urban. An average of 100 arthroscopic shoulder surgeries is performed every year. Enrolment of the study began in February 2020 before the outbreak of the coronavirus (COVID-19) pandemic and is anticipated to continue through 2021.

Prospective participants will be sourced from the surgery waiting list. They will be individually screened by reviewing their health records to determine eligibility. Inclusion and exclusion criteria will be enforced. Patients will be recruited after being admitted to the hospital, just a few hours before their procedure. Baseline evaluation will include a common assessment protocol, baseline spirometry, and US. Since COVID-19 broke out in March, only patients with a negative reverse-transcription polymerase chain reaction (PCR) test result for COVID-19 can be enrolled in the study. They are also required to fill in a COVID-19 triage questionnaire recommended by the European Respiratory Society (ERS) [29]. This trial will comply with all recommendations of ERS and SEPAR for lung function testing [29, 30].

## Assignment of interventions: allocation

### Sequence generation {16a}

Four research teams will participate in this trial: IBPB research physicians (IRPs), assessment research physicians (ARPs), recruitment research physicians (RRPs), and statistical staff (SS). The allocation sequence as per a random number table has been generated using Epidat® software by the SS.

### Concealment mechanism {16b}

The SS will provide the random number table to the IRPs at the beginning of the study. The IRPs, all of whom will be unblinded, in charge of all the IBPB performed in the study. They will be a group of attending anesthesiologists specialized in regional anesthesia. A member of the IRP team will provide the random assignment of each patient to the anesthesiologist in charge of the intervention following a method based on opaque sealed envelopes. Only IRPs will carry out the IBPBs and the administration of the study drug.

### Implementation {16c}

During enrolment, RRPs will be in charge of consecutively including every participant. Once IC has been obtained, ARPs will conduct the spirometry assessments (carried out by a blinded pneumologist), and the US assessments (carried out by a blinded anesthesiologist). Once recruitment is completed, the SS will conduct the study analyses

## Assignment of interventions: blinding

### Who will be blinded {17a}

Due to the nature of the study intervention, IRPs will be the only unblinded research physicians. They will be responsible for the randomization process and for keeping the randomization list. Subjects will be assigned a randomization number and a participant study code. ARPs, who will carry out the US and spirometry assessments, RRPs, care providers, and the trial participants will be kept blinded during the first 24-h post-op.

### Procedure for unblinding if needed {17b}

As mentioned before, IRPs cannot be blinded during treatment allocation. Consequently, a code break procedure will not be necessary. If emergencies happen, they will be managed by unblinded IRPs.

## Data collection and management

### Plans for assessment and collection of outcomes {18a}

All outcome variables will be collected in the data collection form (DCF) and duplicated in the participant medical record (PMRs) by RRPs and ARPs. HDP, the primary endpoint, will be established by US-determined DTR, which provides 93% sensitivity and 100% specificity [23]. Inter-observer and intra-observer reproducibility of diaphragmatic motion in excess of 95% has been reported [31]. Every participant will be evaluated at baseline and after IBPB on both the blocked and the unblocked side of the diaphragm (test and control). Spirometry will be conducted with a certificated portable spirometer with 90% sensitivity and 97% specificity [32, 33]. The test will be performed in the supine and sitting positions for increased the sensitivity and specificity [34]. Postoperative pain will be measured and recorded by using a PCA pump record. PCA provides efficient pain management and increases the patient satisfaction score [35]. The incidence, frequency, and severity of adverse events, according to CTCAE v5.0, will be recorded [25].

Every investigator will be trained in the study interventions. The anesthesiologist in the ARP team received prior diaphragmatic US training for 6 months.

### Plans to promote participant retention and complete follow-up {18b}

Participants may drop out of the study for any reason. The PI may exclude patients to ensure their safety. Withdrawal reasons will be asked, measured, and included in the DCF. Retention will be promoted by organizing explanatory sessions with patients and systematically sending researchers reminders of the meetings. Assessments will be scheduled with ample time so that patients never feel rushed. Non-retention will be distinguished from non-adherence in the monitoring audits.

### Data management {19}

RRPs will enter the (previously coded) eligible patients’ data into the database. After this, IRPs will be responsible for the data throughout the randomization process. Each ARP will have their own DCF where they will enter the data collected during the assessments without breaking the blinding procedures. After the assessments are made, IRPs will collect and secure the completed paper-based DCFs in the study data repository. The PI will check the data in the repository and ensure that the same data has been introduced into the PMRs. He will also conduct a statistical review looking for outlying values, indicative of processing errors. Finally, the PI will enter the data in the database. For promoting data quality, all study data will be stored in duplicate, in the subjects’ PMR, and in the study repository to guarantee maximum traceability. This Protocol only reports on the main aspects of the study. More comprehensive data may be found in the study repository. Access to the repository may be gained by request to the authors. External audits will be conducted to review the study repository every month or every 10 new enrolments. Such audits will be performed by the PI and the monitors appointed by the promoter. The study protocol will be uploaded electronically on the clinicaltrials.gov website, as mentioned in Appendix 1. All data will be stored securely for 25 years at the participating site.

### Confidentiality {27}

This study will be conducted in compliance with the Regulation (Eu) 2016/679 of the European Parliament and of the Council of 27 April 2016 and the Spanish Organic Law of 3/2018 on Personal data protection and guarantee of digital rights [36, 37]. Participants will have the right of access to, rectification of and objection to their data. They can also transfer the data to a third party or request a copy of or cancel their own data during the trial. Participants will at any time be able to contact the hospital’s Data Protection Officer and the promoter. The data collected for the purposes of the study will be coded so that it cannot be traced back to any individual patient. The participants’ identity will not be disclosed to anybody, except to the health authorities if requested, or in case of a medical emergency.

After signing the IC form, all the participants’ data will be coded and identified by an individual trial identification number to maintain participant confidentiality. During the course of the trial, all paper-based patient information will be kept strictly confidential, stored in a separate area under lock and key, accessible only to the PI and the IRPs. All electronical study-related information will be stored on the HUMS network and protected by a firewall at the study site. All records containing names or other personal identifiers, such as locator forms and IC forms, will be stored separately from study records identified by code number. Only the monitors, the PI, the RRPs, and the IRPs will have access rights to the data set. The participants’ study information will not be released outside of the study without their written permission. Anonymized trial data may be shared with other researchers only for research purposes at the authors’ request.

### Plans for collection, laboratory evaluation, and storage of biological specimens for genetic or molecular analysis in this trial/future use {33}

See Item 26b there will be no biological specimens collected.

## Statistical methods

### Statistical methods for primary and secondary outcomes {20a}

Excel (Redmont, USA), IBM SPSS v.22 (Chicago, USA) and Open Epi v3.0.1 (Santiago, Spain) will be used to collect data and conduct analyses. For all analyses, a statistically significant result is assumed if *p* < 0.02. The Bonferroni method will be applied to adjust the overall level of significance for the primary and secondary outcomes.

A descriptive data analysis will be carried out: qualitative variables (sex, surgical side, complications, etc.) will be presented using the frequency distribution of the percentages, and the quantitative variables studied (US and spirometer variables, etc.) will be assessed with the Kolmogorov-Smirnov compliance test (goodness-of-fit test to normal distribution). Central tendency (mean or median) and dispersion (standard deviation or percentiles) indicators will also be given.

To respond to the main hypotheses raised in this study, statistical methods will be carried out. The degree of association between the variables involved will be examined using graphical (scatter diagram) and analytical (simple correlation coefficient) methods. The interpretation of the intensity of the relationship was carried out following the criteria established by Gerstman (2015) and Martinez-González et al. [38, 39]. Concerning bivariate analysis or comparison between two variables (factors), the association between the factors will be investigated using hypothesis contrasting tests. A comparison of proportions with chi-square or Fisher’s exact test will be carried out if both variables compared were qualitative. If one of them was quantitative, a comparison of means will be performed applying Student’s *t* test and ANOVA; if they do not follow a normal distribution, the Mann-Whitney *U* test or the Kruskal-Wallis test will be performed. Likewise, a bivariate correlation (Pearson’s correlation) will be carried out when both variables are quantitative or, if the conditions of application are not fulfilled, a Spearman’s correlation. In some of the cataloged variables, where the case also serves as a control (before-after relationship), comparisons of means will be made for related samples when one of them is quantitative (Student’s *t* test, ANOVA for repeated measurements), and if they do not follow a normal distribution, the Wilcoxon test or the Friedman test will be performed.

### Interim analyses {21b}

An interim analysis will be performed by SS when half of the participants (*n* = 24) have been randomized and have completed the follow-up.

### Methods for additional analyses (e.g., subgroup analyses) {20b}

For multivariate analysis, studying the relationship of each variable controlling for the possible effect caused by third variables, the analysis will be completed using regression models.

### Methods in analysis to handle protocol non-adherence and any statistical methods to handle missing data {20c}

This is a per intention-to-treat study. Outcome data obtained from all participants will be included in the data analysis, regardless of protocol adherence. Missing data will be handled by using a multiple imputation method to complete the data set and its effect will be assessed via sensitivity analysis, as advised by Yuan [40]. If necessary, we will create a set of clinically reasonable imputations for the respective outcome for each dropout. This will be accomplished using a set of repeated imputations created by predictive models based on the majority of participants with complete data. After the imputations are completed, all of the data (complete and imputed) will be combined and the analysis performed for each imputed-and-completed dataset. Our expectation is that very few patients will be lost to follow-up due to current clinical practice nature of the trial intervention. As well, we will report reasons for withdrawal for each randomization group and compare the reasons qualitatively.

### Plans to give access to the full protocol, participant level-data, and statistical code {31c}

See Appendix 1.

## Oversight and monitoring

### Composition of the coordinating center and trial steering committee {5d}

PI (POF) and the other research physicians (JPOL, RGR, IGP, JVC, LOG, LGL, and COM) will be responsible for the design and conduct of the REDOLEV-2019 trial, reviewing the protocol, preparing the DCFs, organizing steering committee meetings, managing the clinical trials office, and publishing study reports. Local organization and responsibilities are explained in items 16b and 16c. The PI, as the lead investigator, will be ultimately responsible for supervising patient identification and recruitment, data collection, and completion of DCFs, along with following up study subjects and their adherence to the study protocol. The steering committee (POF, JPOL, and RGG) will be responsible for approval of the final patient recruitment protocol and for reviewing the progress of the study and, if necessary, introducing changes to the protocol. The committee will meet monthly and before and after the end of the recruitment process. The trial management committee (POF, PMP, and CTG) will supervise the trial, organize the schedule of work, and organize the steering committee meetings. It will also conduct monthly audits of the study venue and report any serious incidents to the health authorities and ethics committee. It will also provide advice to the researchers and be responsible for trial’s master file. Only PMP and CTG will be in charge of establishing the randomization process and attending the meetings of the ethics committee. They will also regularly meet with the PI and the data monitoring committee (DMC) to review the data. There will be no stakeholder or private institutional involvement.

### Composition of the data monitoring committee, its role and reporting structure {21a}

The DMC will be composed by PMP and CTG, who will be required to sign a conflict-of-interest statement. Internal audit meetings will be confidential and devoted to verifying the source documents and adverse events reports. Source documents include DCFs, PMRs, associated reports, and assessment records. The process will be independent from the investigators. It is estimated that interim analyses will be carried out monthly, with an initial and a final analysis. Terms of reference for the DMC are available on request from the IISAragon.

### Adverse event reporting and harms {22}

Adverse events, SAEs, adverse drug reactions, unexpected adverse drug reactions, and serious adverse drug reactions will be defined according to the guidelines for good clinical practice of the European Medicines Agency [41]. The profile of levobupivacaine has been described by the Spanish Agency of Medicines and Medical Devices (AEMPS) [18]. IRPs will determine the relatedness of an incident to the study drug depending on the subject’s medical history, concomitant medications, on whether the incident occurred within the drug’s window of action. In some cases, the incident may be of an unexpected or inexplicable nature.

Every adverse event or reaction will be recorded in the DCF and the PMR. Subjects will be followed up until they are deemed to have overcome or chronified the adverse event.

For secondary outcomes, complications-related variables will be recorded in the operating room, 4 h, 24 h and 30 days after IBPB.

### Frequency and plans for auditing trial conduct {23}

Monitoring visits will be scheduled monthly, or at least every 10 new enrolments.

### Plans for communicating important protocol amendments to relevant parties (e.g., trial participants, ethical committees) {25}

Protocol modifications, SAEs, and other unintended effects of trial interventions or trial conduct will be collecting in the DCF and PMR. They will be duly assessed and managed by the PI and the attending anesthesiologist. They will be reported to the promotor, Ethics Committee, and AEMPS.

### Dissemination plans {31a}

Trial outcomes will be published in (inter) national journals, communicated to anesthesiologist associations, presented at (inter) national congresses, and released to the participating physicians and participants.

## Discussion

Performing an IBPB in patients with respiratory history is a challenge to anesthesiologists as they must administer the lowest possible LA dose to prevent side effects while ensuring proper postoperative analgesia despite potential postoperative respiratory complications. Here, quantity is not always synonymous with quality. Nowadays, US provides enhanced brachial plexus imaging which significantly contributes to the use of as little anesthetic as possible to prevent adverse events. However, this does not always guarantee phrenic block prevention. The contemporary literature suggests that the current performance of the IBPB has improved the HDP prevention, but the safe dose of IBPB remains uncertain.

HDP is usually well tolerated in healthy patients, but in cases of a severe respiratory history, HDP is poorly tolerated, and IBPB is contraindicated. Supraclavicular, suprascapular, or superior trunk blocks have been studied as alternatives to IBPB in arthroscopic shoulder surgery [42–45]. Although supraclavicular block is considered an acceptable choice, clinically similar to IBPB, its incidence of HDP is still up to 59% [46]. On the other hand, superior trunk block has recently achieved similar analgesia, but partial HDP may reach a high incidence of 76% [42]. No regional block has yet shown itself capable of preventing phrenic nerve block and providing adequate postoperative analgesia. Therefore, IBPB is still considered the regional block of choice in shoulder surgery [1].

Few studies in the literature have assessed the performance of IBPB in arthroscopic shoulder surgery by simultaneously evaluating the three main IBPB features: diaphragmatic motion, respiratory function and postoperative analgesia [6, 9, 15]. This is the first RCT in our country to assess them quantitatively using US-determined DTR, spirometry, and PCA analgesia [47]. Similar studies have proposed higher volume and concentration IBPB LA doses than those used in our current practice [15–17]. This study will analyze the performance of one of the smallest volumes used in the literature [15, 17, 48–50]. The methodology used in this study is aimed at increasing the accuracy of HDP diagnosis by including three independent measurements (US, spirometry, and pain). The US assessment will include two different measures: DTR and DE. Every US and spirometric assessment will be made in the sitting and supine positions to assess the influence of the subject’s position on diaphragmatic dysfunction [10, 34, 51]. Moreover, every participant will be their own case-control comparison as diaphragmatic motion will be assessed on both the phrenic blocked side and the contralateral side. Our objective is therefore to document and analyze every benefit and risk of IBPB, removing potential external confounding factors.

A few years ago, chest X-ray and spirometry were the only two tools for diagnosing HDP [3–5]. In the 1990s, US was introduced and the diaphragm thickening fraction was described [52]. Currently, US plays a crucial role in the diagnosis of HDP. B-Mode US is capable of diagnosing HDP with 93% sensitivity and 100% specificity [23]. Inspiratory and expiratory diaphragmatic thicknesses have been measured to predict time to extubation, chronic phrenic paralysis, and pulmonary diseases. Here, they will for the first time be used in an RCT to evaluate the incidence of HDP following IBPB [47]. Diaphragmatic dysfunction has traditionally been diagnosed only to measure null or paradoxical DE [6, 15, 26, 53]. Postoperative analgesia has usually been assessed by a satisfaction scale or by analgesics consumption [53]. This study will add a quantitative analysis of postoperative analgesia by using a PCA pump set for bolus doses to minimize the external influences on pain management and avoid influencing respiratory function. Study interventions will be non-invasive, painless, radiation-free, reproductive, well-tolerated, and exempt from known adverse side effects. The only pharmacological intervention required will be performed as per current practice. Therefore, the study benefits are expected to be greater than any potential complications that could arise.

In summary, our outcomes will contribute to the growing body of evidence in favor of decreasing IBPB LA doses to reduce the incidence of HDP. If the primary and secondary study hypotheses are confirmed, these findings will provide evidence for a safe (low volume) dose of IBPB LA, which will afford adequate postoperative analgesia. This would greatly improve anesthetic management during arthroscopic shoulder surgery. Additionally, the findings of this study will provide critical information regarding patients with a respiratory history undergoing upper extremity surgery for which IBPB has traditionally contraindicated. These patients could therefore benefit from low volume IBPB, which would make general anesthesia unnecessary [6, 16, 17, 47]. This study could help reconsider the contraindications of IBPB and modify current anesthesiologist practice based on the evidence.

## Trial status

The study is currently in the process of recruiting participants. Recruitment commenced on 11 February 2020, was discontinued due to the outbreak of COVID-19, and is due to be completed within 2021. This article is based on version 2.0 uploaded to clinicaltrials.gov.

### Supplementary Information

**Additional file 1.** SPIRIT 2013 Checklist: Recommended items to address in a clinical trial protocol and related documents.

## Data Availability

See Appendix 1.

## Appendix 1

Supplementary data, such as study protocol, IC and DCF of participants, data management details and study data will be uploaded to the study registration section of ClinicalTrials.gov Protocol Registration and Results System website, available at https://clinicaltrials.gov/ct2/show/NCT04385966.

## Appendix 2

### Consent for publication {32}

Informed consent will be available at https://clinicaltrials.gov/ct2/show/NCT04385966.

## Abbreviations

ARP: Assessment research physician
CEICA: Ethics committee of clinical research of Aragon
DCF: Data collection form
DE: Diaphragmatic excursion
DTR: Diaphragmatic thickness ratio
FVC: Forced vital capacity
FEV1: Forced expiratory volume at 1 s
HDP: Hemidiaphragmatic paralysis
HUMS: Miguel Servet University hospital
IBPB: Interscalene brachial plexus block
IC: Informed consent
IRP: IBPB research physicians
IV: Intravenously
LA: Local anesthetic
NRS: Numerical rating scale
PACU: Post-anesthesia care unit
PCA: Patient-controlled analgesia
PI: Principal investigator
RCT: Randomized controlled trial
RP: Research physician
RRP: Recruitment research physicians
SS: Statistical staff
US: Ultrasound
